# Association of Versican Turnover with All-Cause Mortality in Patients on Haemodialysis

**DOI:** 10.1371/journal.pone.0111134

**Published:** 2014-10-29

**Authors:** Federica Genovese, Morten A. Karsdal, Diana J. Leeming, Alexandra Scholze, Martin Tepel

**Affiliations:** 1 Nordic Bioscience, Fibrosis Biology and Biomarkers, Herlev, Denmark; 2 Odense University Hospital, Department of Nephrology, Institute for Molecular Medicine, Cardiovascular and Renal Research, Institute of Clinical Research, University of Southern Denmark, Odense, Denmark; University of Campinas, Brazil

## Abstract

**Objective:**

Cardiovascular diseases are among the most common causes of mortality in renal failure patients undergoing haemodialysis. A high turnover rate of the proteoglycan versican, represented by the increased presence of its fragmentation products in plasma, has previously been associated with cardiovascular diseases. The objective of the study was to investigate the association of versican turnover assessed in plasma with survival in haemodialysis patients.

**Methods:**

A specific matrix metalloproteinase-generated neo-epitope fragment of versican (VCANM) was measured in plasma of 364 haemodialysis patients with a 5-years follow-up, using a robust competitive enzyme-linked immunosorbent assays. Association between VCANM plasma concentration and survival was assessed by Kaplan-Meier analysis and adjusted Cox model.

**Results:**

Haemodialysis patients with plasma VCANM concentrations in the lowest quartile had increased risk of death (odds ratio, as compared to the highest quartile: 7.1, p<0.001), with a reduced survival of 152 days compared to 1295 days for patients with plasma VCANM in the highest quartile. Multivariate analysis showed that low VCANM (p<0.001) and older age (p<0.001) predicted death in haemodialysis patients.

**Conclusions:**

Low concentrations of the versican fragment VCANM in plasma were associated with higher risk of death among haemodialysis patients. A possible protective role for the examined versican fragment is suggested.

## Introduction

Patients with renal failure who undergo haemodialysis have an increased mortality compared to the general population [1], [2]. Cardiovascular diseases are frequently a co-morbidity affecting end stage kidney disease (ESKD) patients [3], [4], and they are the major cause of death in patients on dialysis [5]. A dysregulated equilibrium between extracellular matrix (ECM) formation and degradation characterizes fibrotic disorders, neoplasia and cardiovascular diseases [6]. Renal fibrosis is the pathological process underlying kidney failure, and most of the co-morbidities affecting ESKD patients can be related to the altered matrix turnover [7]. Thus, novel non-invasive biomarkers able to describe the rate of ECM turnover have the potential to be useful instruments to identify patients with a worse prognosis [8].

Versican is a large extracellular matrix proteoglycan whose role in chronic kidney disease (CKD) has only been described to a certain extent [9]. Versican belongs to the family of the large aggregating proteoglycans, and it has been localized in the ECM of many organs, including kidneys [9], [10]. It has a modular structure constituted by a G1 domain at the N-terminal, a glycosaminoglycan (GAG) domain for the attachment of the chondroitin sulphate chains that constitute its carbohydrate fraction, and a G3 domain at the C-terminal end. The G3 domain is further organized in a modular structure containing two EGF-like repeats, a lectin-like subdomain and a complement binding protein (CBP)-like subdomain, which are dedicated to the binding with different ECM components and cytokines [11]. The main protein associated with versican is hyaluronan [12], which interacts with the G1 domain of versican. Moreover versican binds and interacts with elastic fibers, collagen type I, fibronectin and integrins [11]. Versican turnover can be estimated by the amount of fragmentation products released into circulation. A novel neo-epitope specific enzyme-linked immunosorbent assays (ELISA) has been developed to detect a unique fragmentation product generated by the cleavage of matrix metalloproteinases. This fragment of versican variant V0 measurable in plasma (VCANM), has previously been associated with different cardiovascular manifestations [13].

In the present study we tested the hypothesis that versican turnover may be associated with mortality in haemodialysis patients, by measuring plasma levels of VCANM.

## Subjects and Methods

### Participants

We performed an observational cohort study of 364 haemodialysis patients. The study was approved by the local ethics committee (Ethikkommission Free University Berlin, Reference numbers: ek.211-19, ek.Te2.02) and adhered to the declaration of Helsinki. Inclusion criteria were haemodialysis treatment due to end-stage renal disease and written informed consent.

All patients were routinely dialyzed for four to five hours three times weekly using biocompatible membranes with no dialyser reuse. Blood flow rates were 250 to 300 mL/min, dialysate flow rates were 500 mL/min, dialysate conductivity was 135 mS. Blood pressure was measured pre-dialysis in patients in a recumbent position. Pre-dialysis blood samples were taken at study entry. Blood was collected immediately before the start of the haemodialysis session. Clinical and laboratory data included age, gender, medication (use of angiotensin-converting-enzyme inhibitors, ß-blockers, calcium channel blockers, and erythropoietin), body mass index (calculated as weight in kilograms divided by height in meters squared), systolic and diastolic blood pressure, serum urea, serum calcium, serum potassium, and serum phosphorus. 179 patients (49%) died during the 5-years follow up. The causes of death were classified as cardiovascular, infection, cancer, or unknown. Controls consisted of nineteen age- and sex-matched CKD patients not undergoing dialysis. Among these, two patients had stage two CKD (glomerular filtration rate, GFR, according to MDRD: 90–60 mL/min), and seventeen patients had stage three CKD (GFR according to MDRD: 60–30 mL/min).

### Procedures

The neo-epitope peptide generated by matrix metalloproteinase (MMP) degradation of versican (VCANM) was measured in plasma of haemodialysis patients and controls by means of a robust competitive enzyme-linked immunosorbent assays (ELISA) using a specific monoclonal antibody (mAb). The protocols and technical specifications of the assay have already been described [13]. The VCANM mAb was raised against the sequence KTFGKMKPRY, a neo-epitope generated by *in vitro* proteolytic cleavage at the position 3306 by MMP-12. The antibody was selected to recognize specifically the neo-epitope generated after MMP cleavage, and not the total protein.

### Statistical analysis

Continuous variables were expressed as median with interquartile range and compared with Kruskal-Wallis test and Dunn's multiple comparison post-hoc test. Time-to-event analyses were performed using the Kaplan-Meier method. Comparison of survival curves was performed using the log-rank (Mantel-Cox) test. 46 patients (12%) underwent kidney transplantation during the follow up. These patients were censored on the day of transplantation. Univariate and multivariate survival analyses were performed using the proportional hazards regression model. The multivariate model was constructed with backward variable selection, using P<0.05 for variable retention. Statistical analyses were conducted using GraphPad Prism 5.0 (GraphPad Software, San Diego, CA), SPSS for windows (version 15; SPSS, Chicago, IL) and MedCalc (version 12.3.0.0, MedCalc software bvba for Windows).

## Results

### Characteristics of cohort at baseline

A total of 364 haemodialysis patients (240 men and 124 women) with a median age of 67 years (IQR, 56 to 75 years), a median time since initiation of dialysis of 247 days (IQR, 31 to 1142 days), and a median dialysis dose (kt/V) of 1.2 (IQR, 1.0 to 1.3) entered into the study. The primary diseases leading to end stage kidney disease were hypertensive nephrosclerosis in 122 cases (34%), diabetic nephropathy in 118 cases (32%), chronic glomerular nephritis in 30 cases (8%), polycystic kidney disease in 10 cases (3%) and other/unknown in 84 cases (23%). Median plasma VCANM concentration was 0.81 ng/mL (IQR, 0.64 to 0.97 ng/mL). Quartiles of plasma VCANM were used for the survival analysis. Table 1 describes the quartiles and summarizes clinical and laboratory variables stratified according to plasma VCANM quartiles.

**Table 1:** Baseline clinical and biochemical characteristics of haemodialysis patients stratified by plasma VCANM quartiles (Q1, Q2, Q3, Q4).

Characteristic	Q1	Q2	Q3	Q 4	P-value^a^
Number of patients	92	90	92	90	–
VCANM (range; ng/ml)	0.50 (0.20–0.59)	0.70 (0.60–0.79)	0.80 (0.80–0.99)	1.10 (1.00–1.50)	<0.001
Age (years)	68.0 (60.5–76)	64.2 (56–75)	64.3 (52–76)	64.5 (57–72)	0.12
Gender (% Male)	68%	72%	66%	56%	0.06
Dialysis vintage (months)	25.4 (1.0–35.3)	27.8 (1.0–51.7)	26.5 (1.6–43.0)	23.4 (1.0–30.4)	0.88
Diabetes mellitus (%)	50%	31%	62%	60%	0.30
Weight (kg)	71 (60–80)	74 (63–81)	73 (62–79)	75 (64–83)	0.33
Body mass index^b^ (kg/m^2^)	24.4 (21–27.7)	25.3 (22–27.8)	24.6 (21.6–26)	25.6 (22.4–29)	0.13
Systolic blood pressure (mmHg)	131 (114–149)	135 (117–153)	134 (114–150)	134 (117–150)	0.73
Diastolic blood pressure (mmHg)	68 (57–80)	71 (59–80)	70 (58–80)	71 (60–82)	0.35
Hemoglobin (mg/dL)	9.9 (8.7–10.8)	10.0 (9.1–11.4)	10.6 (9.3–11.8)	10.6 (9.3–11.9)	0.02
Leukocytes (10^9^/L)	10.0 (6.6–12.6)	10.0 (6.5–12.8)	8.4 (6.2–9.7)	7.9 (5.7–9.5)	0.002
Platelets (10^9^/L)	243 (173–305)	232 (176–256)	227 (165–271)	248 (187–300)	0.43
Albumin (g/dL)	3.2 (2.6–3.4)	3.2 (2.8–3.6)	3.4 (3.1–3.7)	3.5 (3.1–3.8)	<0.001
High sensitive CRP (mg/dL)	6.7 (2.3–8.0)	4.2 (1.2–4.5)	3.0 (0.75–4.1)	3.8 (0.9–4.7)	<0.001
Urea (mg/dL)	34 (20–36)	33 (20–41)	26 (16–31)	27 (17–35)	0.02
Serum potassium (mmol/L)	4.7 (4.1–5.2)	4.8 (4–5.5)	4.8 (4.3–5.2)	4.8 (4.2–5.4)	0.80
Serum calcium (mmol/L)	2.2 (2–2.4)	2.3 (2.1–2.4)	2.2 (2.1–2.4)	2.2 (2.1–2.4)	0.34
Serum phosphorus (mg/dL)	1.6 (1.2–1.9)	1.7 (1.2–2.1)	1.7 (1.2–2)	1.6 (1.1–2.1)	0.69
Parathyroid hormone (ng/mL)	164 (39–197)	213 (45–272)	199 (43–213)	212 (42–278)	0.96
Serum cholesterol (mg/dL)	142 (108–178)	162 (124–201)	156 (135–181)	166 (137–189)	0.06
LDL-cholesterol (mg/dl)	92 (66–114)	101 (70–129)	98 (79–116)	100 (79–123)	0.55
Dialysis dose (kt/V)	1.2 (1.0–1.3)	1.2 (1.1–1.3)	1.2 (1.0–1.3)	1.2 (1.0–1.3)	0.67
ACE inhibitors (%)	24%	20%	33%	28%	0.23
ß-Blockers (%)	59%	62%	55%	62%	0.87
Calcium channel blockers (%)	23%	29%	38%	29%	0.20
Erythropoietin therapy (%)	48%	42%	62%	45%	0.57

Continuous variables are given as medians (IQR).

a Comparisons between groups were made using Kruskal-Wallis test for continuous variables and Chi-square test for categorical variables.

b Body mass index was calculated as weight in kilograms divided by height in meters squared.

### Plasma VCANM levels are related to mortality

179 patients (49%) died during the 5 year follow up. Death occurred at a median of 201 days (IQR, 63 to 477 days) after study entry. The causes of death were cardiovascular diseases (including sudden death, fatal myocardial infarction, or fatal stroke) in 111 patients (62%), infectious disease (including septicemia) in 35 patients (20%), cancer (any type) in 22 patients (12%), and others/unknown in 11 patients (6%).

**Figure 1:**
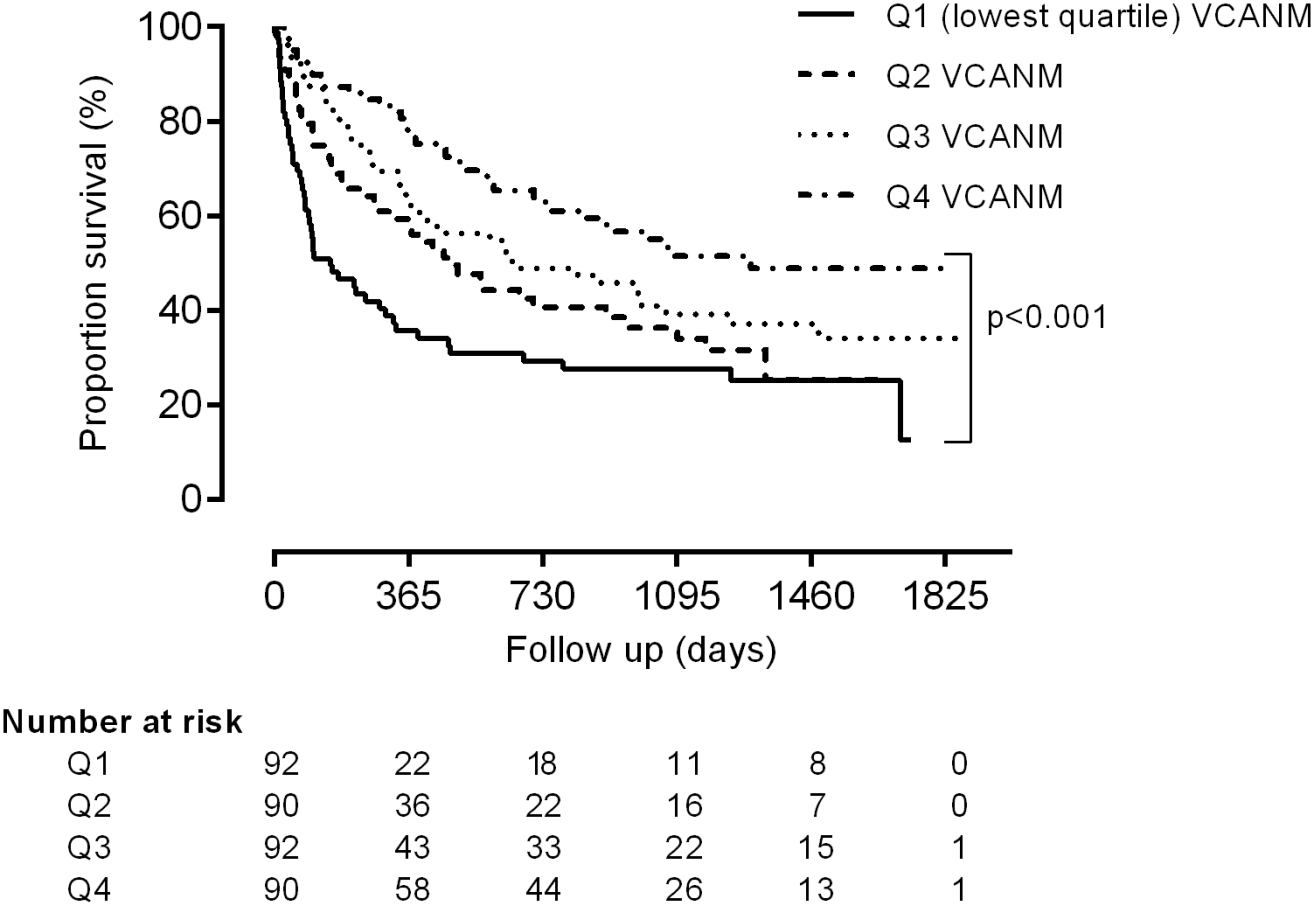
Kaplan-Meier survival curves for haemodialysis patients stratified in quartiles according to plasma VCANM levels.

When patients were stratified according to quartiles of plasma VCANM, the survival rate differed significantly between the different groups (p<0.001). Patients belonging to the lowest VCANM quartile had a survival of 152 days compared to 1295 days for patients with plasma VCNAM in the highest quartile (Figure 1). VCANM was associated to all-cause mortality, and could not distinguish between cardiovascular disease-driven mortality and other mortality causes (data not shown).

**Figure 2:**
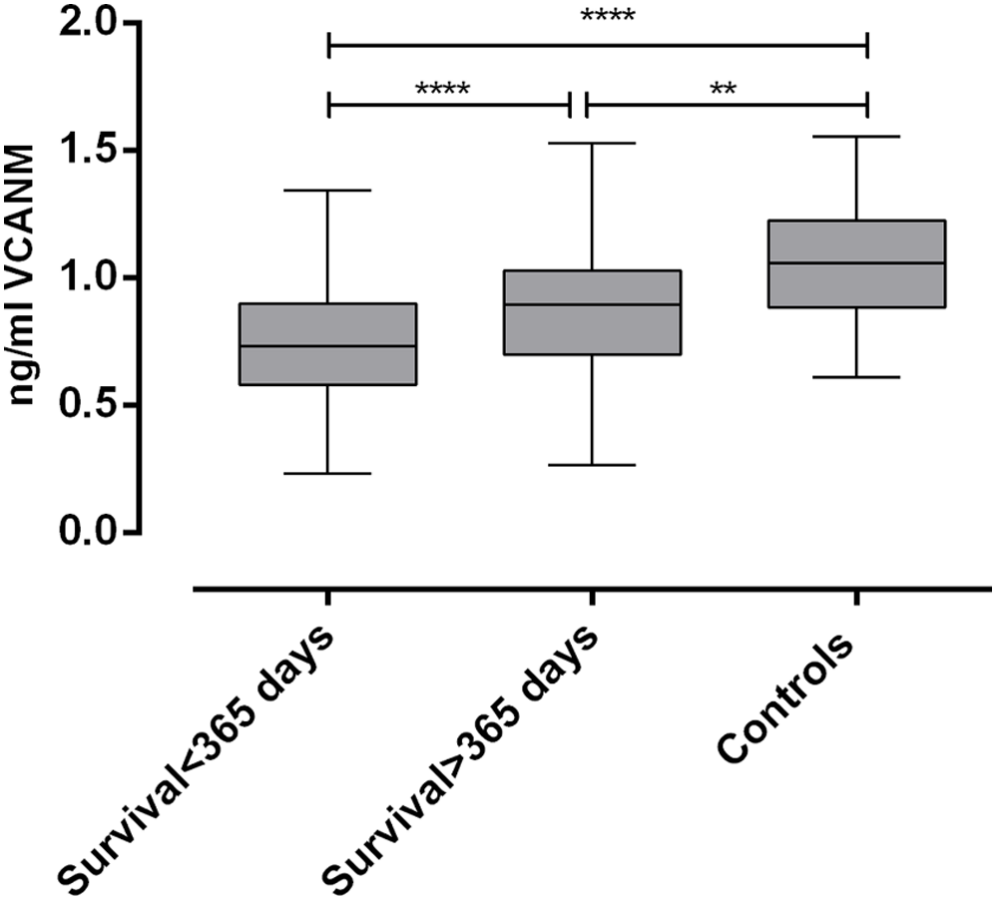
Plasma VCANM levels in haemodialysis patients with survival <365 days and survival >365 days and in the control group. Horizontal line: median; hashed box = IQR; error bars: range of non-outlying values. Significance levels (calculated with one-way ANOVA test, multiple comparison): ** = p<0.01; **** = p<0.001.

**Figure 3:**
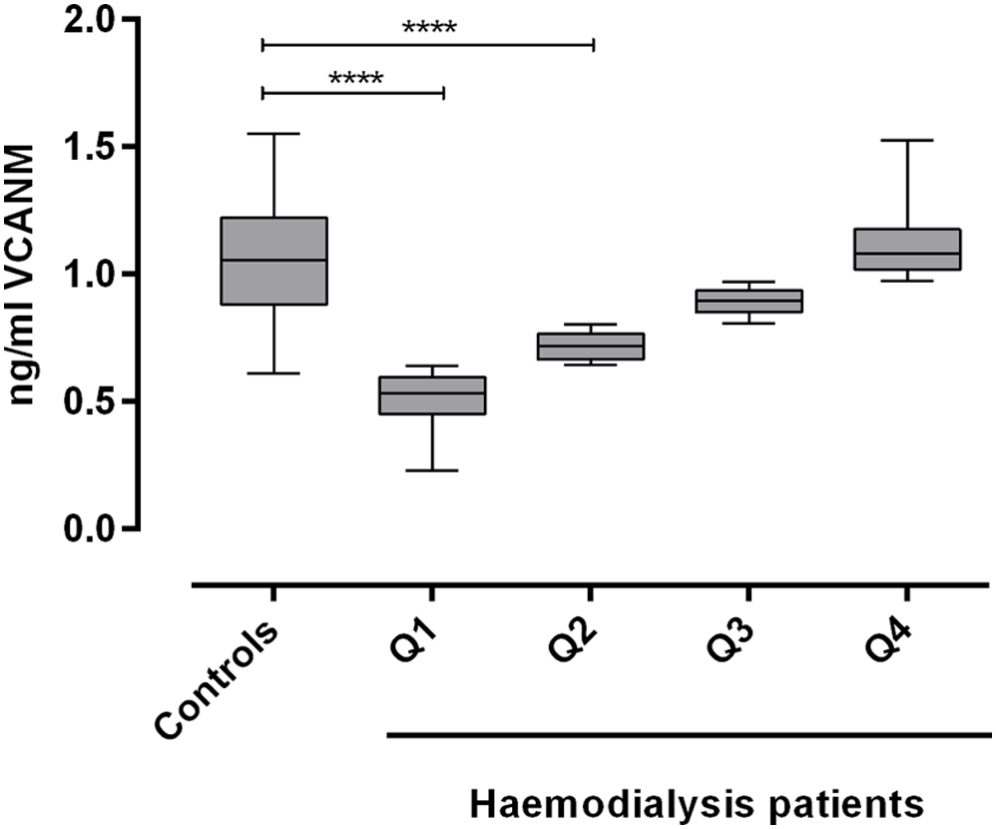
Plasma VCANM levels divided into quartiles in haemodialysis patients and in CKD stage two and three patients (controls). Horizontal line: median; hashed box = IQR; error bars: range of non-outlying values. Significance levels (calculated with Kruskal Wallis test and one-way ANOVA test, multiple comparison): **** = p<0.001.

The differences in survival rate among the different groups appeared to be evident already at early time points, such as 365 days. Therefore a survival time of 365 days was chosen as cut-off and the haemodialysis patients were divided into two groups: one including patients with survival time below 365 days and the other including patients that survived longer than 365 days. The levels of the analyzed marker in these two groups were compared to those of our control group constituted of patients with chronic kidney disease stage two and three (Figure 2). Plasma VCANM levels were highest in the control group, and lowest in the group of patients with short survival. Figure 3 illustrates the concentration levels in the different quartiles in the haemodialysis patient population and in the controls. VCANM levels were the same in controls and in Q4 and decreased significantly in Q3, Q2 and in the lowest quartile Q1.

The univariate Cox regression analysis showed a significant association of VCANM, age, high sensitive CRP and albumin with survival (Table 2). VCANM and age were retained in the model obtained with a multivariate analysis, showing independency from one another. CRP levels were significantly different in the VCANM quartile groups, decreasing with increasing VCANM concentrations (Table 1).

**Table 2:** Univariate and multivariate Cox regression showing the odds for death in haemodialysis patients.

	UnivariateOdds Ratio(95% CI)	P-value	MultivariateOdds Ratio(95% CI)	P-value
VCANM	0.23 (0.12–0.42)	<0.001	0.23 (0.13–0.42)	<0.001
Age	1.06 (1.05–1.08)	<0.001	1.06 (1.05–1.07)	<0.001
Gender (Ref = male)	0.89 (0.66–1.43)	0.31		
High sensitive CRP	1.69 (1.25–2.28)	<0.001		
Albumin	0.67 (0.52–0.86)	0.002		

From the Cox regression analysis it was possible to calculate the odds ratio for an increase of ten years in age (odds = 1.87) and a decrease of 0.5 ng/ml of plasma VCANM (odds = 2.1).

**Figure 4:**
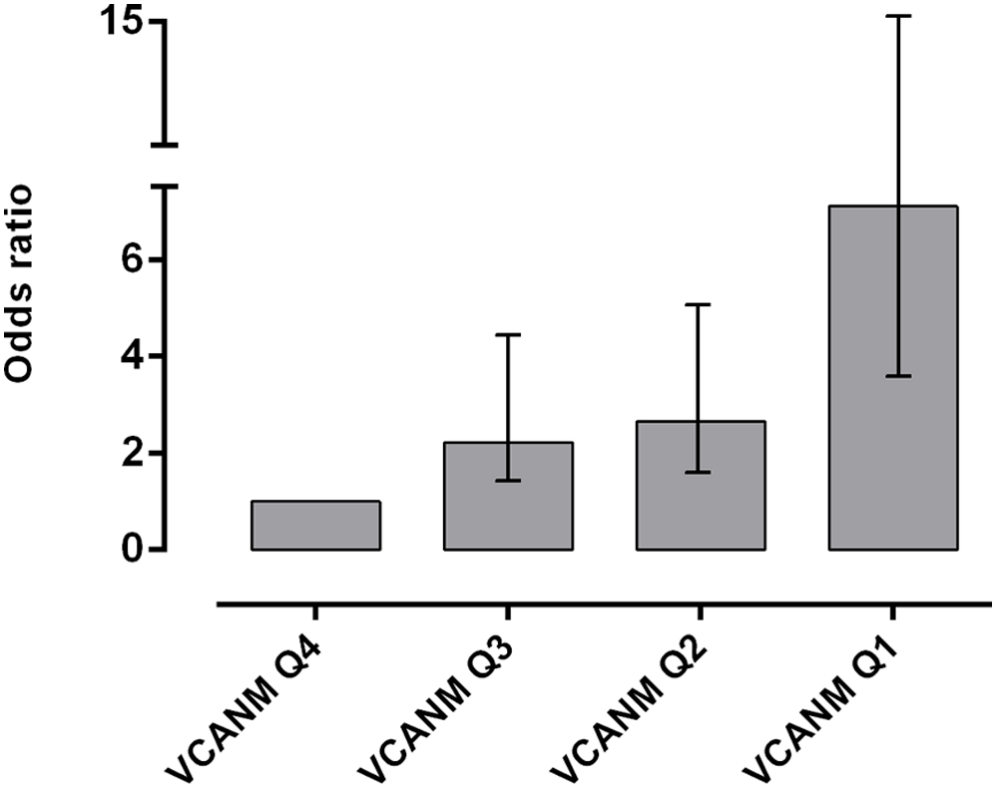
Odds ratio calculated for a cut-off of 365 days in the different VCANM quartiles. Bars: 95% CI.

The odds ratio for the different VCANM quartiles was calculated for death at 365 days as outcome (figure 4). VCANM Q1 showed an odds ratio of 7.1 (using as reference the fourth quartile).

## Discussion

In the present study we have identified a novel marker of mortality in haemodialysis patients.

This marker represents a fragmentation product of versican, an ECM proteoglycans implicated in vascular remodeling [14], and it was previously associated with cardiovascular diseases caused by dysregulated ECM turnover [13]. The VCANM assay measures a pool of fragments of versican long up to 90 aminoacids, which have a common N-terminal end generated by proteolytic cleavage mediated by matrix metalloproteinases. This fragment is localized in the proximity of the protein C-terminal, in the G3 domain, and belongs to the complement binding protein (CBP)-like motif.

Haemodialysis patients with higher VCANM levels showed better outcome. This finding was strengthened by the observation of higher plasma VCANM levels in CKD patients stage two and three (controls) than in haemodialysis patients with shorter survival. Plasma VCANM concentration and age were retained in a multivariate regression model and were independent from each other. Furthermore, a decrease in plasma VCANM levels of 0.5 ng/ml, which corresponded to the median difference between the lowest and the highest plasma VCANM quartile, described a higher risk of death when compared to an increase of ten years of age. The separation between the different survival curves was maximal at early time points. For this reason we chose a cut-off time point of 365 days to calculate the odds ratio. The odds ratio for mortality for patients in the lowest plasma VCANM quartile was seven times higher compared to that for patients in the highest quartile. Interestingly, levels of VCANM were not exclusively associated to cardiovascular mortality, despite the previous findings that versican fragmentation was increased in cardiovascular diseases such as acute myocardial infarction and coronary calcification [13]. This suggests that in ESKD patients VCANM is not a marker for cardiovascular morbidity. The association of lower plasma levels of this specific fragment with a worse outcome might have different possible explanations: an increased fragmentation of versican in patients with a better prognosis may facilitate its clearance. An upregulated versican mRNA expression in the kidney was associated with the degree of histological damage [15], and histological accumulation of versican was observed in the tubulointerstitium of patients with proteinuric nephropathy [9], and in cellular crescents and periglomerular areas of patients with human crescentic glomerulonephritis [16]; therefore the ability to degrade the accumulated proteoglycan might be a factor that ameliorates the disease burden. Versican has been shown to be produced by stromal cells and leukocytes [17]: the correlation between number of leukocytes and VCANM quartiles (as shown in table 1) suggests that versican formation is more elevated than versican degradation during sustained inflammation. It has been demonstrated that complexes of versican and hyaluronan (HA) promote leukocytes adhesion and that a failed incorporation of versican into the ECM blocks monocyte adhesion and reduces the inflammatory response [17]. Therefore when versican is degraded (hence VCANM presence in plasma increases), the formation of the complex versican-hyaluronan is impaired and inflammation is reduced. This hypothesis is further confirmed by the inverse correlation between serum CRP levels and VCANM levels (table 1). Furthermore, as lower levels of VCANM are associated with higher mortality risk, it can be hypothesized that this fragment may have a protective role in end stage kidney disease patients. Such protective functions have been previously observed for other ECM protein fragments, such as endostatin and tumstatin [18], [19]. The paracrine and endocrine functions of the ECM proteins are more and more object of investigation, and the proteins belonging to the matrix are not considered anymore as structural components only. Beside ECM proteins in their native conformation having signaling roles, there are evidences suggesting that ECM proteins that normally don’t exert matricellular functions, gain powerful signaling potential after protease cleavage [6], [20]. As of today, versican has only been seen as a therapeutic target [21], but the finding that high levels of its degradation product VCANM are associated with longer survival introduces the novel concept of its potential as therapeutic *per se*, which, however, needs to be carefully examined. Further investigations are needed to confirm whether VCANM is not only a fragment generated by proteolytic cleavage of versican, but has also paracrine or endocrine functions.
